# Miocene diversification of a golden‐thread nanmu tree species (*Phoebe zhennan*, Lauraceae) around the Sichuan Basin shaped by the East Asian monsoon

**DOI:** 10.1002/ece3.6710

**Published:** 2020-08-26

**Authors:** Jian‐Hua Xiao, Xin Ding, Lang Li, Hui Ma, Xiu‐Qin Ci, Marlien van der Merwe, John G. Conran, Jie Li

**Affiliations:** ^1^ Plant Phylogenetics and Conservation Group Centre for Integrative Conservation Xishuangbanna Tropical Botanical Garden Chinese Academy of Sciences Kunming China; ^2^ University of Chinese Academy of Sciences Beijing China; ^3^ Department of Landscape Architecture Guangdong Eco‐engineering Polytechnic Guangzhou China; ^4^ Center of Conservation Biology Core Botanical Gardens Chinese Academy of Sciences Mengla China; ^5^ Research Centre for Ecological Resilience Australian Institute of Botanical Science The Royal Botanic Garden Sydney Sydney NSW Australia; ^6^ Australian Centre for Evolutionary Biology and Biodiversity (ACEBB) Sprigg Geobiology Centre (SGC) School of Biological Sciences The University of Adelaide Adelaide SA Australia

**Keywords:** climate change, demographic history, *Phoebe zhennan*, population divergence, RAD‐seq, the intensification of East Asian monsoon

## Abstract

Understanding the role of climate changes and geography as drivers of population divergence and speciation is a long‐standing goal of evolutionary biology and can inform conservation. In this study, we used restriction site‐associated DNA sequencing (RAD‐seq) to evaluate genetic diversity, population structure, and infer demographic history of the endangered tree, *Phoebe zhennan* which is distributed around the Sichuan Basin. Genomic patterns revealed two distinct clusters, each largely confined to the West and East. Despite sympatry of the two genomic clusters at some sites, individuals show little or no evidence of genomic introgression. Demographic modeling supported an initial divergence time between the West and East lineages at ~15.08 Ma with further diversification within the West lineage at ~7.12 Ma. These times largely coincide with the two independent intensifications of the East Asian monsoon that were initiated during the middle (Langhian) and late Miocene (Messinian), respectively. These results suggest that the Miocene intensification phases of the East Asian monsoon played a pivotal role in shaping the current landscape‐level patterns of genetic diversity within *P. zhennan*, as has been found for the interspecific divergence of other subtropical Chinese plants. Based on isolation‐by‐distance and species distribution modeling, we hypothesize that *P. zhennan* followed a ring diversification which was facilitated by the Sichuan Basin acting as barrier to gene flow. In situ and ex situ conservation management plans should consider the results obtained in this study to help secure the future of this beautiful and culturally significant endangered tree.

## Graphical abstract

The Miocene intensification phases of the East Asian monsoon, combined with geographic distance, played a pivotal role in the evolution shaping the plants species of subtropical China.

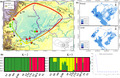

## 1 INTRODUCTION

It is well known that global climate change and geography have profound impacts on population divergence and speciation (Schluter & Pennell, 2017; Zou, Yang, Doyle, & Ge, 2013) and the climate of Asia has experienced a series of drastic changes since the Miocene. These changes include the transformation from a zonal climate pattern (a broad, W–E dry belt across China) to a monsoon‐dominated pattern (early Miocene: ~20–15 Ma; Clift, Wan, & Blusztajn, 2014; Guo et al., 2008); the disappearance of typical subtropical aridity (Sun & Wang, 2005); and the onset of desertification in Central Asia (~22 Ma; Guo et al., 2008). These major climate transitions were caused mainly, if not exclusively, by the uplift of the Qinghai–Tibetan Plateau (QTP) (Tada, Zheng, & Clift, 2016), with further ongoing and rapid uplift of the QTP linked to intensifications of the East Asian monsoons at about 15 Ma, 9–7 Ma and 3.6–2.6 Ma (An, Kutzbach, Prell, & Porter, 2001; Sun & Wang, 2005).

During the Pleistocene, the monsoon system interacted with glacial–interglacial cycles, producing a more variable monsoon climate (An et al., 2001; Sun & Wang, 2005). Previous studies have shown that the dynamic history of the East Asian monsoon played a major role in organism evolution in the QTP and adjacent regions (see review in Favre et al., 2015), largely by controlling the hydrological cycles (Tada et al., 2016). Most of these earlier studies focused on interspecific evolutionary process, and few have emphasized the role of past historical events and evolutionary factors on population divergence and speciation since the Miocene; a period that witnessed some of the most significant climate changes and orogenesis in Asia (An et al., 2001; Guo et al., 2008; Zachos, Dickens, & Zeebe, 2008).

Geographic isolation can facilitate divergence and speciation events, because barriers such as distance, water bodies, and mountains can impede gene flow and drive genetic differentiation through allopatric or parapatric speciation (Pritchard, Stephens, & Donnelly, 2000; Winger & Bates, 2015). Southwest China, particularly the mountains surrounding the Sichuan Basin (e.g., the Hengduan Mountains to the west, the Daba Mountains to the north and northeast, and the Wuling and Dalou Mountains to the south and southeast), has been identified as a global diversity hotspot, due in part to its mild monsoon climate and complex topography (Myers, Mittermeier, Mittermeier, Da Fonseca, & Kent, 2000). This region has been recognized as a “plant museum” for relictual angiosperms and other seed plants by providing long‐term ecological and environmental stability (López‐Pujol, Zhang, Sun, Ying, & Ge, 2011). Several phylogeographic studies suggested that geographic and climatic events during the late Miocene (11.2–5.3 Ma) played important roles in the development of intraspecific lineages in southwest China (Qi et al., 2012; Sun et al., 2014; Zhang et al., 2013). However, only a few of these studies have focused specifically on the role of the Sichuan Basin as driver of speciation and for most taxa and the interactions between geography and climate as far as back as the late Miocene remain understudied.

*Phoebe zhennan* (Lauraceae) is a diploid (2*n* = 24), dioecious tree up to 30 m tall which produces purple‐brown drupes and grows in subtropical evergreen broad‐leaved forests (EBLFs) of China (Li, Li, & van der Werff, 2008). Chinese *Phoebe* L. species are insect‐pollinated, and the seeds are dispersed primarily by frugivorous birds (Li et al., 2008; Li, Liu, Ma, Zhang, & Xu, 2018). *Phoebe zhennan* is the major source of the well‐known wood “Golden‐thread nanmu,” which is extremely valuable due to its durability, unique special fragrance, and attractive golden color. This timber has been used for the manufacture of coffins and the construction of palaces and furniture for over a thousand years (Lan, 1994; Zhang, 2010). As a result of its desirability, the current sporadic distribution of the around the Sichuan Basin is largely due to habitat loss and logging and most extant populations consist of fewer than 70 individuals (Ding, Xiao, Huang, & Li, 2015; Fu, 1992) and the species is listed as endangered in the Chinese Plant Red Book (Fu, 1992).

Although previous studies of *P. zhennan* mainly focused on its habitat characteristics (Li et al., 2013), seedling biology (Xie et al., 2017), and species delimitation from other “Nanmu” tree species (Ding, Xiao, Li, Conran, & Li, 2019), little is known about its evolutionary history. An earlier investigation of genetic diversity using amplified fragment length polymorphisms (AFLPs) in *P. zhennan* showed low genetic diversity and significant divergence, apparently linked with topography in southwestern China (Gao et al., 2016). However, that study was inconclusive about the demographic history of *P. zhennan*, or whether past climate changes may have had a strong impact on its evolutionary history, especially in relation to the observed “ring‐species” pattern of variation.

Restriction site‐associated DNA sequencing (RAD‐seq) is a popular approach for ecology, evolutionary population genomic and conservation genomics of nonmodel species (with or without a prior genomic resource), which can generate genome‐wide single nucleotide polymorphisms (SNPs) cost‐effectively and quickly (Baird et al., 2008; Andrews, Good, Miller, Luikart, & Hohenlohe, 2016; Parchman, Jahner, Uckele, Galland, & Eckert, 2018). Additionally, RAD‐seq leverages the site frequency spectrum (SFS), providing a powerful tool for demographic history inference (Excoffier, Dupanloup, Huerta‐Sánchez, Sousa, & Foll, 2013; Gutenkunst, Hernandez, Williamson, & Bustamante, 2009; Hotaling et al., 2018; Sousa & Hey, 2013).

Therefore, we applied SNPs markers generated through RAD‐seq to investigate genetic differentiation patterns of *P. zhennan* around the Sichuan Basin and infer its demographic history to understand processes that may have led to extant patterns. We addressed the following two questions in regard to the population genomics and the demographic history of *P. zhennan*: (a) How is genetic diversity distributed and what is the population structure? (b) Can we identify the factors that impacted on the current distribution of genetic diversity within the species?

## 2 MATERIALS AND METHODS

### 2.1 Sample collection and RAD‐seq sequencing

A total of 72 *P. zhennan* trees were sampled from across 12 sites around the Sichuan Basin (Table 1), with voucher specimens processed and deposited in the Herbarium of Xishuangbanna Tropical Botanical Garden (HITBC). Fresh leaves were dried in silica gel and stored at −20°C before processing for DNA extraction. Genomic DNA was extracted using a modified CTAB protocol (Rogers & Bendich, 1989), and quality checked by electrophoresis on 1% agarose gels and normalized to a DNA concentration of 30 ng^−mL^. RAD‐seq libraries were prepared following the protocol described in Baird et al. (2008). Briefly, genomic DNA was digested using the restriction enzyme EcoRI (5′‐G*AATTC‐3′). Fragmented DNA was then ligated to Illumina sequencing adaptors (Illumina Biotechnology Company) containing sample‐specific barcode sequences, followed by PCR. Library preparation and pair‐end sequencing (85 bp) were carried out by the Beijing Genomics Institute using an Illumina Hiseq 2500.

**TABLE 1:** Site characteristics, sample sizes (*n*), and habitat information for 12 populations of *P. zhennan* across its distribution in Sichuan Basin and adjacent areas

Code	Location	Longitude (E)	Latitude (N)	Elevation	*n*
QCS	Qingchengshan, Sichuan	103°34′17.11″	30°53′57.66″	745 m	6
QL	Huojing, Sichuan	103°11′56.24″	30°20′46.43″	744 m	6
MS	Mingshan, Sichuan	103°12′13.74″	30°14′19.11″	592 m	6
EMS	Emeishan, Sichuan	103°26′5.58″	29°33′50.42″	535−1,120 m	12
SF	Shuifu, Yunnan	104°19′9.94″	28°34′4.52″	519 m	6
YJ	Yanjin, Yunnan	104°22′20.85″	28°13′59.72″	685 m	6
CN1	Changning, Sichuan	105°0′42.27″	28°40′42.51″	276 m	3
CN2	Changning, Sichuan	104°58′45.57″	28°27′46.13″	509 m	3
JY	Jiangyang, Sichuan	105°19′0.01″	28°49′31.26″	469 m	6
XY	Xuyou, Sichuan	105°31′24.46″	28°19′7.19″	351 m	6
YC	Yongchuan, Chongqing	105°53′14.80″	29°33′47.98″	427 m	6
TL	Tongliang, Chongqing	106°5′48.29″	29°35′17.00″	479 m	6
R1	–	103°44′47.67″	29°06′10.79″	–	–
AX	Anxian, Sichuan	104°20′24.00″	31°35′24″	–	–
PW	Pingwu, Sichuan	104°33′3.6″	31°27′3.6″	–	–
CK	Chengkou,Chongqing	108°37′48″	31°49′12″	–	–
WX	Wuxi, Hubei	109°37′48″	31°22′12″	–	–

Note

For RAD‐seq sequencing, we collected 12 populations. However, AX, PW, CK, and WX sites were only used for species distribution modeling (SDM) because we cannot collect samples in these collection areas and adjacent regions. The R1 point is used to estimate “ring distances” among populations for isolation‐by‐distance analysis (IBD).

### 2.2 De novo assembly and SNPs calling

We performed de novo assembly and single nucleotide polymorphisms (SNPs) using the STACKS 1.35 pipeline (Catchen, Amores, Hohenlohe, Cresko, & Postlethwait, 2011; Catchen, Hohenlohe, Bassham, Amores, & Cresko, 2013). Prior to analysis, poor quality reads (phred scores less than 30), reads with possible adapter contamination, and those lacking restriction sites (using *process_RADtags*) were removed. Remaining reads were then assembled into loci in “*ustacks*” with a maximum distance between stacks of *M* and minimum read depth of *m*. The loci were clustered further into “*cstacks,*” with mismatches allowed between samples. To increase coverage depth and maximize loci, a minimum read depth of 6 (*m* = 6) and a maximum distance between stacks of 2 in “*ustacks*” (*M* = 2), and 6 mismatches in “*cstacks*” (*n* = 3) in STACKS was set. After finishing de novo assembly, SNPs calling and genotyping were performed using “populations” model in STACKS. Loci with 100% presence among populations (*p* = 12) were retained, whereas loci with >25% missing data within populations were removed (*r* = 75%) to maximize shared SNPs across individuals. The first SNP per locus and SNPs with minor allele frequencies (MAF) ≥ 0.05 were retained. After completing SNP calling, those remaining with a Hardy–Weinberg equilibrium (HWE) *p*‐value less than .05 were also removed using VCFtools v4.0 (Danecek et al., 2011). PGDspider v2.02 (Lischer & Excoffier, 2012) was used subsequently for file conversion to program‐specific formats.

### 2.3 Population genomic analyses

Three datasets were generated for the population genetic analyses: (a) the full matrix with all polymorphic loci for genetic summary statistics; (b) a subset consisting of 16,210 SNPs with HWE filtering based on the full matrix for pairwise *F*_ST_, AMOVA, principal component analysis (PCA), and historical relationships; and (c) the minimum dataset consisting of 5,000 SNPs dataset extracted randomly from 16,210 SNPs for Bayesian clustering in the STRUCTURE v2.3.4 (Pritchard et al., 2000). Because there are often difficulties in resolving genomic datasets using STRUCTURE, Rodriguez‐Ezpeleta et al. (2016) suggested that a dataset with up to 5,000 SNPs is an optimal analysis size to capture the ancestral group.

Genetic summary statistics for RAD‐seq genomic data, including percentage of polymorphic sites (%*P*), observed and expected heterozygosity (*H*_obs_ and *H*_exp_), nucleotide diversity (*π*), and inbreeding coefficients (*F*_IS_), were estimated using *populations* in STACKS for all populations (10) with more than six samples. Pairwise *F*_ST_ values were calculated using Arlequin v3.5.2 (Excoffier & Lischer, 2010), with 10,000 permutations. Hierarchical AMOVA analysis of molecular variance (AMOVA) was implemented based on our assessment the hierarchical population structure (*K* = 2 and *K* = 3, see results and discussion) in order to quantify genetic variation partitioning across the different sampling levels.

Genetic structure was inferred using Bayesian clustering in STRUCTURE based on the minimum dataset (e.g., the randomly selected 5,000 SNPs dataset). An admixture model with no priori information for sampling location was utilized to determine whether the number of clusters (*K*) ranged from 1 to 13. For each value of *K*, 10 independent simulations were conducted with a burn‐in length 100,000, followed by 300,000 iterations. The best *K* value was identified based on the successive change of LnP(D), Evanno's delta *K*, and individual assignment probabilities (Evanno, Regnaut, & Goudet, 2005), as implemented in STRUCTURE HARVESTER v0.6.93 (Earl & Vonholdt, 2012). Results were plotted using DISTRUCT (Rosenberg, 2004), and population structure investigated further with PCA using the R package *adegenet* (Jombart, 2008).

We used TreeMix v1.13 (Pickrell & Pritchard, 2012) to address historical relationships between populations. The VCF file was converted to a frequency file that could be transformed to a TreeMix file using the *plink2treemix* script. The number of migration events was tested by starting at zero and adding one by one until the residual plot stopped improving. The resulting maximum‐likelihood (ML) tree and residual plot were visualized in R and illustrated in combination with the STRUCTURE results.

### 2.4 Isolation‐by‐distance (IBD) analysis and cline analysis

An isolation‐by‐distance pattern was examined using the R package *vegan* (Oksanen, Kindt, Legendre, & O'Hara, 2006). A mantel test implementing *vegan* with 1,000 permutations was used to detect significant correlations between genetic and geographic distances, with *F*_ST_/(1 − *F*_ST_) values used to represent genetic distance. Geographic distances among the sites with “straight‐line distances” and “ring distances” were calculated in the R package *geosphere* (Hijmans, Williams, & Vennes, 2011). The ring distances assume dispersal routes following the ring distribution, instead of straight lines across intervening uninhabitable areas. For example, initial analyses suggested that there is a genetic “barrier” between QCS and QL with little evidence of gene flow across the gap. Based on predicated suitable habitat and the ring‐shaped distribution, we therefore used one reference point (R1) to approximate the ring distance between sites. All distances between Western and eastern sites were estimated by the sum of the distances from Western sites to R1 and then R1 to eastern sites.

Geographic cline analysis was conducted to detect any sudden changes in population genetic composition or “hybrid zones” along the distribution ring using the R package *hzar* (Derryberry, Derryberry, Maley, & Brumfield, 2014). Clines were defined by distance from site QCS in one dimensional space, following an approximate circle around the basin and using the first principal component analysis axis (PC1) as a proxy of genetic variation. Five cline models with different fitting tails (none fitted; left only; right only, mirror tails; both tails estimated separately) and the minimum and maximum genetic variation (pMin, pMax) fixed‐to‐observed values were tested. To facilitate model comparison, a null cline model was also established, which assumes that genetic variation is independent of any clines. The best model was decided upon Akaike's information criterion (AIC) scores. Finally, the maximum‐likelihood clines and summary statistics were extracted from the best‐fit model.

### 2.5 Demographic history inference

Fastsimcoal2.6 (Excoffier et al., 2013) was implemented to infer the spatial and temporal demographic history of *P. zhennan* using the site frequency spectrum (SFS). Populations were grouped into three clusters based on STRUCTURE, PCA, and ML results in order to capture the main characteristics of the population histories for the East group (JY, XY, and CN1), West1 group (EMS), and West2 group (SF and YJ populations). The dataset used for demographic analysis was generated from the dataset 2 (16,210 SNPs), but using only a SNP with the lowest allele frequency per RAD‐seq locus. The sample sizes of gene copies for the East, West1, and West2 groups were 30, 24, and 24, respectively.

Twenty demographic models were tested (Figure S1; detailed in Appendix S1), and detailed model schematics are provided in Appendix S1. Briefly, models included scenarios specifying: (a) two divergence events for all possible topologies of the three genetic clusters (models 1–9); (b) models where admixture between two existing genetic clusters created the third (models 10–18); and (c) trifurcation models where extant genetic clusters emerged simultaneously from a common ancestor (models 19–20). For all models, the potential for bidirectional gene flow was varied both historically and recently.

The fastsimcoal2 analyses followed an initial set of model selection runs, with comparisons of maximum observed and expected likelihoods to select the best‐fit model, then subsequent parameter estimation through simulation of new SFS for the best‐fit model, followed by parametric bootstrapping (Method S2; detailed in Appendix S1). Fifty independent parameter estimations were performed to achieve the maximum composite likelihood of the joint fold‐SFS, in which parameterized the simulation sample from prior and the parameter estimation was optimized through 40 cycles of a conditional maximization algorithm. The point estimates were selected from the run with the highest maximum composite likelihood, with confidence intervals of parameter estimates obtained by 100 parametric bootstrapping runs from simulated SFS of the parameter estimates.

### 2.6 Species distribution modeling (SDM)

SDM was carried out in Maxent v3.4.1 (Elith et al., 2011; Phillips, Anderson, & Schapire, 2006) to predict the potential and suitable distribution range of the species and also to investigate whether Sichuan basin may have served as a barrier potentially facilitating ring diversification. A total of 47 species occurrence records were obtained from herbarium collections and specimen records (Table S1; detailed in Appendix S1). Nineteen bioclimatic variables (Table S2; detailed in Appendix S1) for both Last Glacial Maximum (LGM, ~22,000 years ago) and the present period (~1950–2000) were obtained from the WorldClim database (http://www.worldclim.org/; Hijmans, Cameron, Parra, Jones, & Jarvis, 2005). To avoid multicollinearity, the initial variables were filtered based on the results of Pearson's correlation analysis (Synes & Osborne, 2011). For each highly correlated variable pair (Pearson's *r* ≥ 0.7), the variable that gave a higher value in the regularized gain and the percent contribution to the Maxent model was retained (Method S1; detailed in Appendix S1).

Maxent was configured with 75% of species presence data for training and 25% for testing data, and sampling procedure was replicated 50 times. The area under the curve (AUC) was estimated to test the accuracy of the model prediction (Method S1; detailed in Appendix S1). The distributions of these periods were then plotted on a Chinese map using a geographic information system as implemented in the software ArcGIS 10.4 (Environmental Systems Research Institute, Inc.)

## 3 RESULTS

### 3.1 RAD‐seq dataset

After filtering reads with an average quality score below 30, the number of reads for each individual (85 bp/reads) ranged from 4,096,070 to 16,201,221 (with an average reads per sample of 9,780,563) with a GC content of 37.9%. After de novo assembly and SNP calling, there were 43,906 preliminary assembled loci, 31,834 polymorphic loci, and 59,288 SNPs (the full dataset had 1.86 SNPs for each locus), corresponding to the full matrix. After SNP filtering, the minor dataset yielded 16,210 loci and 16,210 SNPs.

### 3.2 Genomic diversity

Based on 31,834 polymorphic loci, the average percentage of genomic polymorphic sites (%*P*) for each population was 1.11 and ranged from 0.72 to 1.95 per population. Private alleles were present in all populations varying from 19 (YJ population) to a maximum of 3,435 (EMS population). Observed heterozygosity (*H*_O_) across all populations ranged from 0.227 to 0.252 for each population (average 0.243), and expected heterozygosity (*H*_E_) across all populations ranged from 0.118 to 0.186 (average 0.158). Expected heterozygosity for each population was consistently lower than observed heterozygosity, and nucleotide diversity (*π*) across all populations ranged from 0.0039 to 0.0051 (average 0.0048) for each population (Table 2).

**TABLE 2:** Genomic diversity statistics calculated from the full dataset (31,834 polymorphic loci data and 41,720 SNPs)

Samples	%*P*	PA	RAD loci				SNPs			
			*H*_O_	*H*_E_	*π*	*F*_IS_	*H*_O_	*H*_E_	*π*	*F*_IS_
QCS	1.16	49	0.0071	0.0043	0.0048	−0.0042	0.227	0.138	0.152	−0.135
QL	0.81	114	0.0079	0.0040	0.0044	−0.0064	0.252	0.128	0.141	−0.203
MS	1.28	1,068	0.0076	0.0046	0.0051	−0.0045	0.247	0.151	0.165	−0.146
EMS	1.95	3,435	0.0076	0.0058	0.0061	−0.0033	0.243	0.186	0.196	−0.107
SF	1.19	821	0.0075	0.0049	0.0053	−0.0042	0.25	0.163	0.179	−0.142
YJ	0.8	33	0.0078	0.0039	0.0044	−0.0062	0.26	0.132	0.145	−0.208
JY	1.2	922	0.0070	0.0043	0.0047	−0.0041	0.233	0.143	0.157	−0.136
XY	1.26	1,076	0.007	0.0048	0.0053	−0.0036	0.234	0.16	0.176	−0.119
YC	0.76	327	0.0075	0.0038	0.0041	−0.0061	0.249	0.126	0.139	−0.202
TL	0.72	19	0.0071	0.0036	0.0039	−0.0058	0.234	0.118	0.130	−0.19
Overall	1.11	‐	0.0074	0.0044	0.0048	−0.0048	0.243	0.145	0.158	−0.159

Note

Statistics are provided for *P. zhennan* sampling localities. Observed heterozygosity (*H*_O_), expected heterozygosity (*H*_E_), nucleotide diversity (*π*), and the inbreeding coefficient (*F*_IS_) were calculated for RAD loci and only variable positions (SNPs), respectively. Additional abbreviations include %*P* = percent of polymorphic sites, PA = private alleles.

Based on these *π* and *H*_E_ values, within‐population genomic diversity levels were rank‐ordered as EMS > SF > XY > MS > QCS > YJ > QL > YJ > YC > TL (Table 2). In addition, the southern and southwestern populations showed the higher genomic diversity whereas the populations at the tails of the chain (QL and TL population) had lower genetic genomic diversity (%*P* and *π*, Table 2; Figure 4), with a decreasing trend around the southwest to west, as well as from the southeast to west of the ring. The inbreeding coefficient (*F*_IS_) was negative and ranged from 0.0064 to 0.0033, indicating low levels of inbreeding in populations. The summary statistics (*H*_O_, *H*_E_ and *F*_IS_) showed the same trend when using all nucleotides (Table 2).

### 3.3 Population genetic structure

Pairwise *F*_ST_ among populations, using the minor dataset, ranged from 0.034 to 0.454 (average 0.258), indicating interpopulation differentiation. In particular, the short straight‐line distances between QCS and the Western and southwestern populations (SF, YJ, EMS, MS, and QL) relatively high pairwise *F*_ST_ values (between 0.220 and 0.377) suggested population differentiation in this region, while the *F*_ST_ values between QCS and the southern and southeastern sites (JY, CN1, XY, TL) were negative, with slightly negative *F*_ST_ values reported as zero (Table 3). Similarly, pairwise *F*_ST_ values between QL and TL, the junction between the end of the Western sites (QL) and southeastern sites (TL) was particular high (*F*_ST_ = 0.446) (Table 3). An unexpected relatively high pairwise *F*_ST_ between YC and TL sites (20.5 km apart) was 0.444.

**TABLE 3:** Pairwise ***F***_ST_ values and gene flow (Nm) among 12 populations of *P. zhennan* calculated for 16, 210 SNPs

	QCS	QL	MS	EMS	SF	YJ	CN1	CN2	JY	XY	YC	TL
QCS	–											
QL	0.377	–										
MS	0.291	0.265	–									
EMS	0.220	0.259	0.193	–								
SF	0.278	0.317	0.248	0.185	–							
YJ	0.389	0.421	0.362	0.297	0.034	–						
CN1	−0.131	0.415	0.321	0.250	0.299	0.418	–					
CN2	0.351	0.385	0.305	0.049	0.277	0.400	0.400	–				
JY	−0.076	0.375	0.293	0.225	0.279	0.387	−0.133	0.345	–			
XY	−0.038	0.321	0.239	0.170	0.226	0.337	−0.068	0.278	−0.036	–		
YC	0.325	0.403	0.329	0.257	0.315	0.419	0.413	0.379	0.372	0.320		
TL	−0.068	0.446	0.366	0.295	0.351	0.454	−0.157	0.433	−0.069	−0.004	0.444	–

Note

Average: 0.258; Nm = 0.719 (According to Nm = (1 − ***F***_ST_)/4 * ***F***_ST_).

**FIGURE 1:**
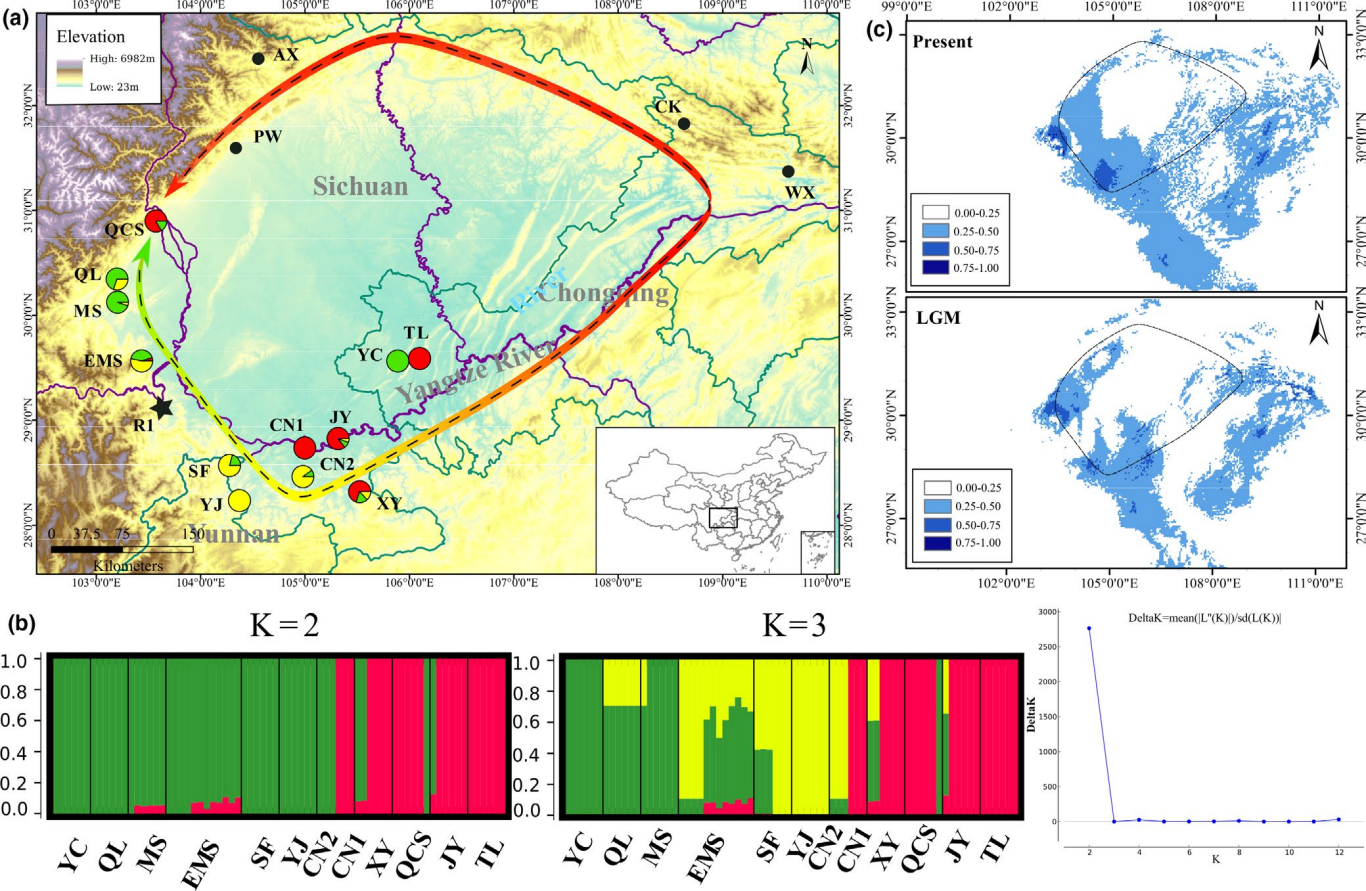
(a) Map of Sichuan Basin with sampling site of *P. zhennan*. Sample sites are color‐coded according to their genetic clusters (*K* = 3). The black points represent specimen records from the Chinese Virtual Herbarium (CVH, http://www.cvh.ac.cn/) and are not subjected to sequencing. The black pentagram represents reference point, which is used for estimating “ring distance” between sites for isolation‐by‐distance analysis (IBD). (b) Individual assignment probability barplots from the genetic clustering analysis using the STRUCTRUE. *K* = 2 is the best fit for our data. (c) Potential distributions as probability of occurrence for *P. zhennan* in southwest China at present (~1950–2000) (top) and at last glacial maximum (LGM, ~22,000 years; bottom). The dash line to present the outline of the Sichuan basin. The scale on the bottom left indicates the probability of occurrence. Maps are generated using the software ArcGIS 10.4

The STRUCTURE analyses using the minimum dataset revealed a clear peak of Evanno's delta of *K* = 2, suggesting that there were two distinct genetic clusters around the Sichuan Basin (Figure 1b). Under this model, most samples from the Western sites (QL, MS, EMS, SF, YJ), a southern site (CN2), and a southeastern site (YC) formed one (West) cluster; whereas the other southern sites (JY, CN1, XY), a southeastern site (TL), and a Western site (QCS) formed the second (East) cluster. Hierarchical clustering analysis at *K* = 3 showed that the Western cluster could be divided further into two distinct genetic clusters (West1 and West2), with the SF and YJ populations clustering separately from the remainder. The East cluster showed no clear subdivisions or evidence of differentiation among samples.

**FIGURE 2:**
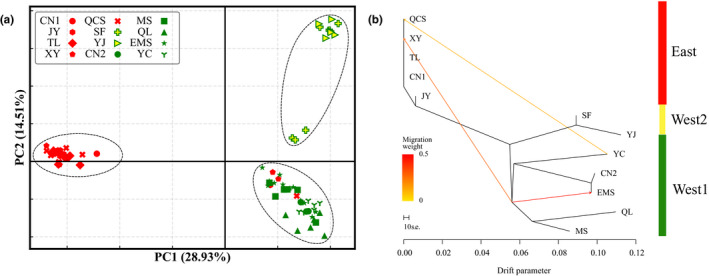
Results of the PCA and TreeMix analyses for 12 populations of *P. zhennan*, showing three groups and indicating gene flow from West to East. (a) Principal component analysis (PCA), with the proportion of the variance explained being 28.93% for PC1 and 14.51% for PC2. Red‐colored shapes represent east cluster, green‐colored shapes represent west1 cluster, and yellow‐colored shapes represent west2 cluster. (b) Admixture graph of genetic groups with three migration events for 12 populations obtained with TreeMix

Consistent with STRUCTURE results, PCA of the minor dataset (16,210 SNPs) revealed three distinct clusters for the 12 populations corresponding to the West1 (QL, MS, EMS, CN2 and YC), West2 (SF and YJ), and East (JY, CN1, XY, TL and QCS) lineages (Figure 2a). The first and second principal components (PC1 and PC2) explained 28.93% and 14.51% of the total variation, respectively. These clusters are congruent with clusters inferred with the *K* = 3 results in STRUTURE (Figures 1b and 2a).

The maximum‐likelihood tree for the 12 populations (Figure 2b) also largely reiterated the relationships inferred with the cluster analysis and explained 97.08% of the population variance relatedness. When migration events were added to the tree, there were three migration edges and the model explained 99.08% of the relatedness variance. These migration edges indicated three admixture events: (a) from the ancestor of the East group into EMS (*w* = 47%), (b) from the ancestor of the East group into XY (*w* = 29%), and (c) from YC into QCS (*w* = 12%; Figure 2b; detailed in Appendix S1). Within the broad West group, there was strong evidence for admixture, supported by visually apparent residuals (Figure S2; detailed in Appendix S1), which is consistent with previous clustering analysis.

Despite the different sampling levels, the AMOVA revealed significant differentiation among groups identified by STRUCTURE and most the variation (*K* = 2:67.3% and *K* = 3:68.72%, respectively) occurred within populations (Table 4). The AMOVA results showed significant genetic differentiation in the two major groups (East and West groups) and among populations (*F*_CT_ = 0.174; *F*_ST_ = 0.327). The AMOVA results also suggested that there is strong genetic isolation between the East, West1, and West2 groups (*F*_CT_ = 0.207; Table 4).

**TABLE 4:** Analysis of molecular variance among populations of *P. zhennan* around the Sichuan basin using 16, 210 SNPs

Model	Fixation index*	Variance (%)
Model A	AG: ***F***_CT_ = 0.174	17.35
	AP: ***F***_SC_ = 0.185	15.32
	WP: ***F***_ST_ = 0.327	67.33
Model B	AG: ***F***_CT_ = 0.207	20.66
	AP: ***F***_SC_ = 0.134	10.61
	WP: ***F***_ST_ = 0.313	68.72

Note

Individuals were grouped according to two different models and subjected to a hierarchical analysis of variance. Model A: groups correspond to *K* = 2 in STRUCTURE analysis; Model B: groups correspond to *K* = 3 in STRUCTURE analysis. All fixation index values are significant at *p* < .0001.

* AG, among groups; AP, among populations within groups; WP, within populations.

### 3.4 IBD and cline analysis

**FIGURE 3:**
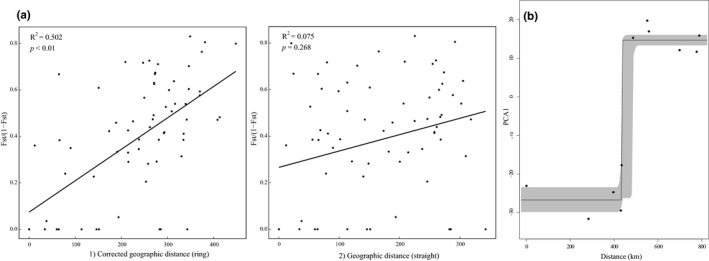
(a) (Left) Correlation between genetic distance (*F*_ST_/1 − *F*_ST_) and ring geographic distance. (Right) Correlation between straight genetic distance and geographic distance. The ring distances approximate the potential gene flow connection among the chain of populations around the Sichuan Basin. (b) Results from cline analysis. The *x*‐axis is the distance from QCS following the potential suitable habitat surrounding the Sichuan Basin. The *y*‐axis is PC1 from principal component analysis

A significant IBD relationship among the populations was also detected, but Mantel tests detected a nonsignificant positive correlation between the *F*_ST_/(1 − *F*_ST_) values and the straight‐line geographic distances between all sites (*r* = 0.075, *p* = .272; Figure 3a). However, when adjusted ring distances were used, the correlations became both significant and much stronger (*r* = 0.502, *p* < .001; Figure 3a). Cline analysis (Figure 3b) found that the “none fitted tail” model represented the best‐fit, with the estimated cline center located at 435 km (434.84–442.10 km around the XY site) and the estimated cline width was 1.37 km (0.74–17.67 km).

### 3.5 Demographic history

Results of the fastsimcoal2 analysis showed that the best‐supported scenario was model1, with an initial divergence between the East and West lineages and subsequent divergence between the West1 and West2 sublineages, with bidirectional gene flow (Figure 5; Table S3). These results provided strong support for a historical bottleneck, followed by subsequent expansion for all lineages.

The divergence time between the East and West lineages was estimated at 1,005,501 (95% CIs: 944,631–1,066,371) generations ago, corresponding to 15.08 Ma (95% CIs: 14.17–15.99 Ma with a generation time of 15 years). The second divergence time between West1 and West2 sublineages occurred 474,808 (95% CIs: 462,466–487,149) generations ago, corresponding to 7.12 Ma (95% CIs: 6.94–7.31 Ma). The current effective population size (*Ne*) was 78,864 (95% CIs: 75,006–82,723) for West1, 722,318 (95% CIs: 704,964–739,672) for West2, and 757,511 (95% CIs: 733,244–781,778) for East. The ancestor of all lineages (N_Anc_all) was 850,047 before divergence at T_A0_ (Table 5), and the ancestor of West1 & West2 lineages (N_Anc_w12) was 34,972 before divergence at T_12_. Parameter estimation showed that ancestral effective population sizes were higher than those of current ones. Moreover, the East lineage had the largest effective population size, whereas West1 had the smallest (East > West2 > West1). Migration probability from West1 into West2 (1.00e^−05^, 95% CIs: 9.55e^−06^–1.05e^−05^) was the highest, but the reverse was low (2.63e^−08^, 95% CIs: 1.41e^−08^–3.85e^−08^), as was migration from West1 into East (3.70e^−6^, 95% CIs: 3.57e^−06^–3.83e^−06^) and the reverse (2.02e^−6^, 95% CIs: 1.86e^−06^–2.19e^−06^). Overall migration probabilities between West2 and East were very low, but historical migration probabilities were not asymmetrical, with migration from ancestors of West lineage into East at 4.16e^−07^ (95% CIs: 2.52e^−07^–1.09e^−06^) and the reverse at 1.89e^−07^ (95% CIs: 1.22e^−07^–2.56e^−07^) (Table 5).

**TABLE 5:** Parameter estimates from Fastsimcoal2 analyses

Parameters	Estimates	95% CIs	Notes
N_cur_W1	78,864	75,006–82,723	Current effective population size of West1 group
N_cur_W2	722,318	704,964–739,672	Current effective population size of West2 group
N_cur_East	757,511	733,244–781,778	Current effective population size of East group
N_Anc_all	850,047	791,362–908,731	Ancestral for ancestor of three groups effective population size
N_Anc_w12	34,972	29,632–40,313	Ancestral for ancestor of west groups effective population size
T_1_	1,005,501	944,631–1,066,371	Divergence time between East and the ancestor of West groups
T_2_	474,808	462,466–487,149	Divergence time between West1 group and West2 group
M12	1.00e−05	0.96e−05–1.05e−05	West1=>West2 group migration rate (2Nm) at time T_12_
M21	2.63e−08	1.40e−09–3.26e−09	West2=>West1 group migration rate (2Nm) at time T_12_
M01	3.70e−06	3.57e−06–3.82e−06	East=>West1 group migration rate (2Nm) at time T_12_
M10	2.02e−06	1.86e−06–2.19e−06	West1=>East group migration rate (2Nm) at time T_12_
M20	3.13e−09	1.98e−09–4.28e−09	West2=>East group migration rate (2Nm) at time T_12_
M02	5.06e−08	2.92e−08–7.22e−08	West1=>East group migration rate (2Nm) at time T_12_
M0_w12	1.89e−07	1.22e−07–2.55 e−07	East=> Ancestor of west group migration rate (2Nm) at time T_A0_
Mw12_0	4.16e−07	2.52e−07–1.09e−06	Ancestor of West group =>East group migration rate (2Nm) at time T_A0_

Note

Demographic parameters are reported for the M1 model passing both the AIC and the goodness‐of‐fit criteria. Confidence intervals were generated by parametric bootstrapping.

### 3.6 Species distribution model (SDM)

The potential and suitable distribution ranges of *P. zhennan* predicted by SDM are shown in Figure 1c, with AUC values >0.9 implying that the performance of Maxent model is outstanding. The current most suitable habitat is clearly ring‐shaped, encircling the Sichuan Basin, with the majority of the central basin being unsuitable. During the LGM, there were fewer potentially suitable habitats and there was lower connectivity between habitat patches (Figure 1c), with potential distributions shifted upslope to areas such as the Qionglai and Wumeng mountains and the Chuang Dong valley. The best‐fit model for the extant conditions was influenced largely by temperature seasonality (Bio4), max temperature of the warmest month (Bio5), precipitation of the warmest quarter (Bio18), and precipitation of the coldest quarter (Bio19), suggesting that these are potential limiting factors for the species current distribution.

## 4 DISCUSSION

This study provides population genomic analyses of the endangered, golden‐thread wood (*P. zhennan*) based on RAD‐seq data, allowing investigation of its distribution patterns and evolutionary history around the Sichuan Basin and the processes that may have influenced their formation. Coalescent modeling supported initial divergence into East and West lineages, with subsequent divergence within the West sublineages, likely coinciding with the intensifications of the East Asian monsoon during the middle and late Miocene, respectively. This supports the hypothesis that the development of the East Asian Monsoon notably impacted intraspecific divergence and population dynamics. Further investigations using the ring distance of the IBD patterns suggested that extant *P. zhennan* populations displayed a ring‐shaped distribution around the Sichuan Basin, supporting the hypothesis that the Sichuan basin may have served as biogeographic barrier (Qiao et al., 2018), facilitating ring diversification.

### 4.1 Population genomic diversity and diversification around the basin

A pattern of low level genomic diversity within populations, coupled with most variation occurring within populations and significant genetic differentiation between groups (Table 3), was observed in *P. zhennan*. There are several possible explanations for these observed patterns. First, fragmentation may lead to the prevalence of genetic drift over gene flow and increase differentiation among populations by isolation. This was supported by the low Nm (0.719; Table 3). Moreover, if Nm < 1, genetic drift can lead to population differentiation; whereas when Nm > 1, then there is generally little differentiation among populations and migration is more important than genetic drift (Wright, 1949). Higher *H*_O_ than *H*_E_ is expected from obligate outcrossing taxa such as dioecious plants, and this will increase within genetic diversity (Nazareno, Bemmels, Dick, & Lohmann, 2017; Stojanova, Šurinová, Zeisek, Münzbergová, & Pánková, 2020).

Second, compared to wind‐pollinated species, pollen of insect‐pollinated species (such as most species in Lauraceae, Li et al., 2018; Rohwer, 1993) disperses over shorter distances, resulting in limiting gene flow and increased genetic differentiation. For example, the pollinators of *Neolitsea sericea* (Blume) Koidz. generally only travel about 4 km (Chung, Chung, Oh, & Epperson, 2000), so short‐distance pollination could similarly be a factor influencing the population structure of *P. zhennan*. Lastly, the patterns observed here may be a consequence of past genetic bottlenecks, or genetic drift associated with small effective population sizes and founder effects (Birky, Fuerst, & Maruyama, 1989). This bottleneck model was supported by demographic history inference, which showed that the ancestral population size for *P. zhennan* was larger than any of the descendant ones (Table 5; Figure 5), with descendant lineages apparently showing evidence of past bottlenecks and/or expansion.

Similar to previous AFLP results (Gao et al., 2016), the clustering analyses (PCA and STRUCTURE) were congruent and indicated that all individuals clearly group into two main groups. These groups were defined largely by geography (West and East), but with a few exceptions, with the West and East (including south and southeast populations) groups showing limited connectivity (Figures 1b and 2). Interestingly, at two sites (YC and QCS), individuals were sampled that showed no signs of genomic introgression but which clustered with samples from the divergent geographic group (Figures 1b and 2). The PCA and ML analysis results revealed two distinct sublineages within the West region: West1 and West2 (Figures 1b and 2; also congruent with *K* = 3). Levels of gene flow as suggested by estimated *F*_ST_ among these sublineages indicated higher levels of genetic connectivity between these clusters. Cline analysis revealed sharp genetic changes between clades and slow genetic changes within clades (Figure 3b), although this may be the result of missing data in the northwestern Sichuan Basin.

### 4.2 The dynamics of the East Asian monsoon shaped a middle Miocene diversification of *Phoebe zhennan*

Several processes likely contributed to the patterns of divergence inferred with genomic data. The intensification of the East Asian monsoon has previously been proposed as a process that shaped intra species diversification. Our demographic analysis suggested that the timing of divergence between the two major groups occurred during the early middle Miocene (15.08 Ma), with subsequent divergence within the West clade during the early late Miocene (7.12 Ma). Similar Miocene‐aged patterns of deep intraspecific divergence have also been reported for two Chinese tree species: *Cyclocarya paliurus* (Batal.) Iljinsk. (~16.69 Ma; Kou et al., 2016) and *Tetracentron sinense* (~9.6 Ma; Sun et al., 2014).

Different lines of evidence provide support for the divergence time estimates within *P. zhennan* during the middle Miocene. First, fossils attributed to *Phoebe* have been reported from the Paleocene of the Northeast India (e.g., *P. sublanceolata*; Bhattacharyya, 1983), extending there to the Pliocene (Khan & Subir, 2014). *Phoebe* fossils have also been reported from the early Miocene to late Pliocene of China (Huang et al., 2016; WGPC, 1978). Second, divergence timing in demographic inference is influenced strongly by the settings used for Fastsimcoal2 with SFS data. We chose the model process after 50 replications for every candidate model and also considered the influence of different mutation rates, with the results almost always identical for the origin of *P. zhennan* (see supplementary information). Third, it takes 10–15 years from seed to first flowering in *P. zhennan* (http://www.nfgrp.cn/), so a generation time of 15 years is both relatively conservative and similar to other long‐lived Lauraceae (e.g., *Lindera obtusiloba* Blume) (Ye et al., 2017).

This study also provides an opportunity to explore the impact of Miocene climate change and tectonic events on the phylogenetic structure of plants in subtropical China. The divergence time between the East and West clades during the early middle Miocene corresponds closely to the initial intensification of the East Asian monsoon at ~15–13 Ma (Sun & Wang, 2005). Although temporal coincidence cannot be regarded as proof of a causal relationship between the evolution of an organism and the dynamic of the East Asian monsoon, it is still reasonable to link the divergence between the East and West lineages for several reasons. First, the SDM analyses indicated that the survival of *P. zhennan* strongly depends on the availability of precipitation and the climate in subtropical southwestern China was apparently drier and cooler before the Miocene, meaning that *P. zhennan* was unlikely to be present until after the Asian monsoon system was established around the time of the Oligocene–Miocene boundary (~23 Ma) and again after the initial intensification of the East Asian monsoon at ~15 Ma.

Second, the initial intensification of the East Asian monsoon in the middle Miocene triggered the intraspecific level diversification of lineages inhabiting subtropical EBLFs in East Asia (Yu et al., 2017). For example, divergence in *Cyclocarya paliurus* coincides with the first intensification of the East Asian monsoon, which provided the necessary humid climatic conditions for it to survive in southwestern of China (Kou et al., 2016). Similarly, the distribution of the fern genus *Lepisorus* (J. Sm.) Ching correlates with precipitation brought by the summer monsoon and its radiation matches the initial intensification of the East Asian monsoon ~15 Ma (Wang et al., 2005). Precipitation parameters are also the most important explanatory factors for more than half of the relict plant genera in central China, especially flagship taxa such as *Cercidiphyllum japonicum* Siebold & Zucc. (Cercidophyllaceae; Qi et al., 2012), *Metasequoia glyptostroboides* Hu & W. C. Cheng (Cupressaceae; Huang et al., 2015), and the ornamental herb *Primula obconica* Hance (Primulaceae; Yan et al., 2012).

The second divergence event seen between the West1 and West2 sublineages seems to have occurred during the late Miocene, coinciding with the second phase of the East Asian monsoon intensification at ~9–7 Ma (An et al., 2001; Harrison, Copeland, Kidd, & Yin, 1992). Similar diversification patterns have also been reported for other Asian plants, with a phylogeographical study of *Tetracentron sinense* Oliv. (Trochodendraceae) in southwestern China revealing intraspecific species divergence ~9.6 Ma (Sun et al., 2014). Deep genetic divergences are also seen along the Eastern Margin of the Yungui Plateau, a well‐known geological boundary (CASPG, 1984), and these may have been influenced by periods of rapid uplift of the QTP. Additionally, many subtropical EBLF trees (including *P. zhennan*) are very sensitive to winter temperatures and will die at temperatures below −10°C (Sakai, 1979; Woodward & Williams, 1987). Such a north–south distribution shift might therefore be explained in part by late Miocene global cooling (Zachos et al., 2008). Together, these results suggest that climatic changes around the Sichuan Basin since the middle Miocene could have been important drivers of the current observed genetic differentiation seen in *P. zhennan* and other subtropical trees in East Asia.

**FIGURE 4:**
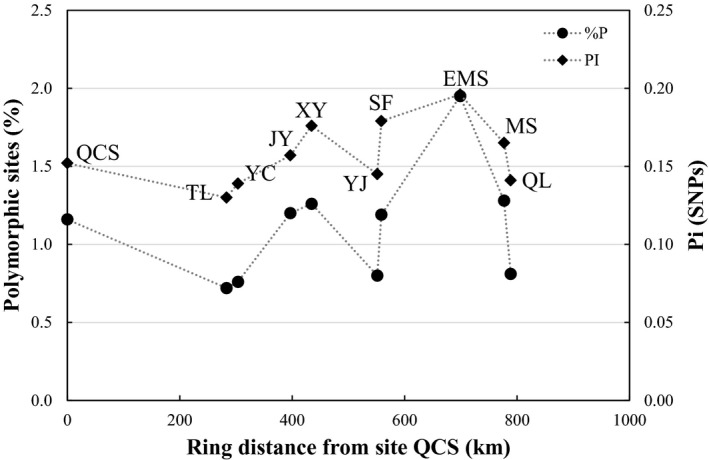
Distribution of genetic diversity across the ring. The *x*‐axis is the distance from QCS following the potential suitable habitat surrounding the Sichuan Basin. The left *y*‐axis is percentage of polymorphic sites for each population, and right y‐axis is nucleotide diversity (Pi)

In addition to climatic events that may have facilitated the deep divergences seen within *P. zhennan,* the Sichuan Basin may have also facilitated the ring like expansion and isolation‐by‐distance pattern currently observed in the species. Further sampling along the Northwestern and North eastern edge of the Sichuan Basin would help to explore this possible ring diversification, but we highlight our results here to propose the hypothesis. SDM analyses indicated that suitable habitat for *P. zhennan* forms a largely continuous ring round the margins of the Sichuan Basin, whereas it is largely absent in the central Basin region (Figure 1c). Interesting, the IBD analysis also produced a very strong correlation between genetic distance and the ring distances (Figure 3a). This correlation was only slightly smaller than that reported for the Caribbean *Eurphorbia titymaloidies* L. (*r* = 0.68; Cacho, Monteverde‐Suarez, & Mcintyre, 2019), the only other plant to date with molecular support for the “ring‐species” model, where a ring of populations encircles an area of unsuitable habitat (here the Sichuan Basin) and exhibits continuous and gradual transitions between intermediate geographically contiguous forms (Alcaide, Scordato, Price, & Irwin, 2014; Wake, 2001). Additionally, in the populations collected around the Basin, we found a decrease in genetic diversity from southern populations toward the southeast and Western populations, that is, the two ends of the ring (Figure 4).

**FIGURE 5:**
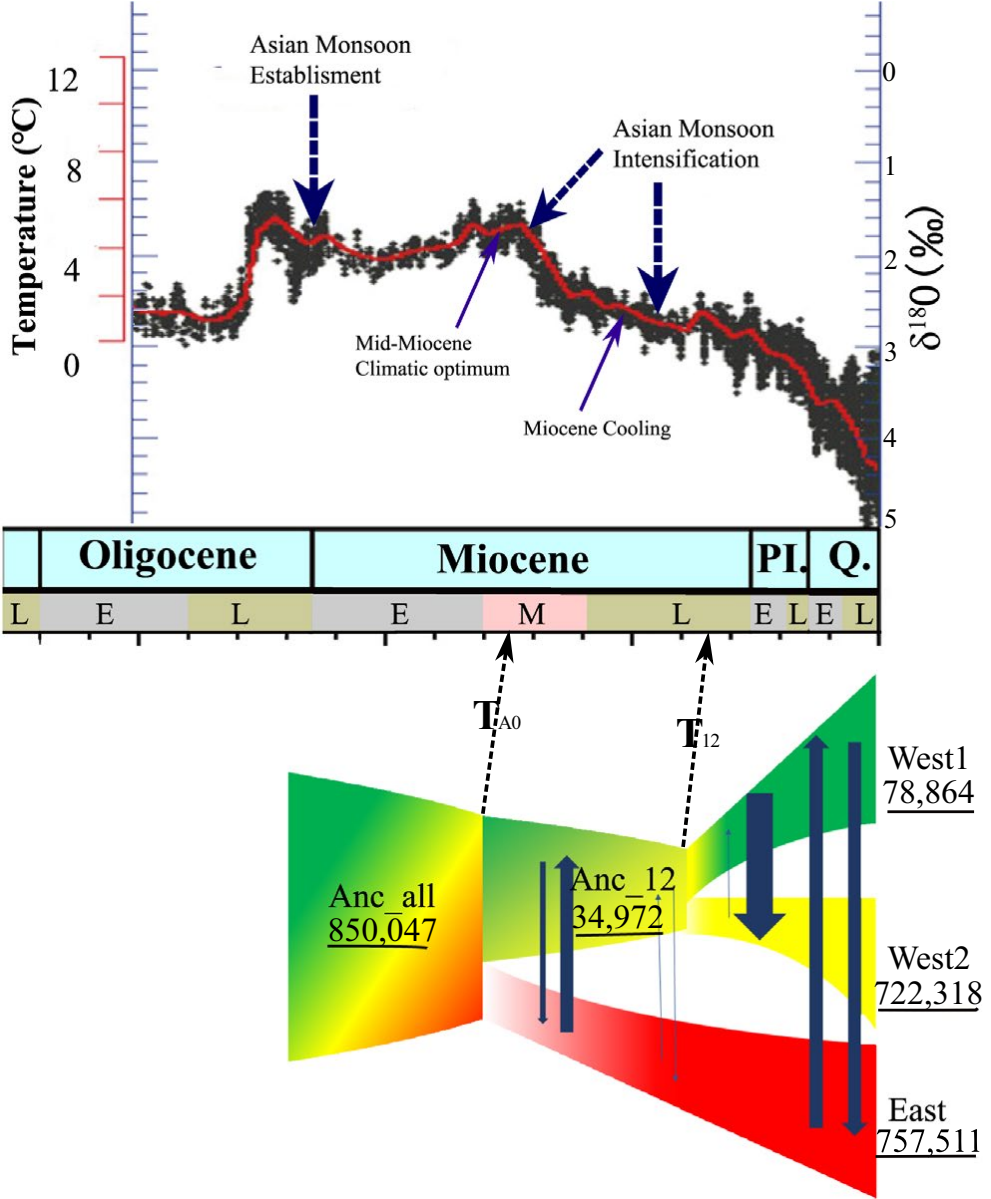
A schematic of the best‐fit demographic model (Model 1) using fastsimcoal2 for *P. zhennan*. The T_A0_ represents the divergence time occurred at the middle Miocene (~15.08 Ma) between west and east lineages. The T_12_ represents the divergence time occurred at late Miocene (~7.12 Ma) between west1 and west2 lineages. The numbers with underline represent the effective population size (Ne), respectively. Blue arrows width indicates the relative migration probability based on a log scale. Ancestral coloration was arbitrarily chosen to simplify visualization. The lineages are colored with the same STRUCTURE analysis

Topographic modeling has suggested that the Sichuan Basin is an excellent candidate for the geographic facilitation of ring speciation (Monahan, Pereira, & Wake, 2012). Moreover, several species from disparate evolutionary lineages display circular distribution patterns around the Basin, including amphibians (e.g., the green odorous frog, *Odorrana margaretea* (Liu, 1950) (Ranidae); Qiao et al., 2018), herbs (*Dysosma versipellis*; (Hance) M. Cheng (Berberidaceae); Guan, Fu, Qiu, Zhou, & Comes, 2010), and trees such as *P. zhennan* S. Lee & F. N. Wei (Lauraceae) (Ding et al., 2015), *Tetracentron sinense* Oliver (Trochodendraceae) (Sun et al., 2014), and *Euptelea pleiosperma* Hook.f. & Thomson (Eupteleaceae) (Wei, Sork, Meng, & Jiang, 2016). Qiao et al. (2018) found that the ring diversification displayed by the green odorous frog around the Sichuan Basin was associated with geographic barriers that isolated refugial populations during the Ice Ages. Therefore, the history of the climate and vegetation in the Sichuan basin might also play an important role in the patterns of genomic diversity and population structure of *P. zhennan*, but this requires further investigation.

### 4.3 Conservation management

To facilitate the long‐term conservation and sustainability of a species, it is widely recognized that it is crucial to protect and encourage within‐species genetic diversity, as genetic diversity increases the ability of a species to evolve and adapt in the long run to a changing environment. Processes such as habitat fragmentation or loss and selective logging can have detrimental effects on both the survival of a species and its genetic diversity, and these factors are already impacting on *P. zhennan*. Golden‐thread wood has been part of Chinese culture for many thousands of years (Li, Jin, & Xiang, 2004), and conservation is both valuable for biodiversity and cultural heritage. Here, we identified three genetically divergent population groups within the species, each with its own unique pattern of diversity and connectivity shaped by past climatic fluctuations.

We identified relatively low levels of gene flow between the two main genomic groups that likely diverged in the Miocene (Figure 1b, *K* = 2). The lack of genetic assimilation between the two groups is particularly evident in populations QCS and YC. Both populations are geographically close to populations belonging to the other genomic group, yet show little sign of genetic introgression (Figures 1b and 2). This has important applications for management, as it suggests that these groups maintain their identities and do not readily outcross. While we can only speculate on the origin of the disjunct YC and QCS populations, three causes are often cited: (a) human introduction; (b) long distance dispersal; and (c) isolation after species range contractions, due to either climatic or anthropogenic reasons (Meeus, Honnay, & Jacquemyn, 2012). The QCS population is located in Fengshui forest that is found near an ancestral temple, and the YC population is located in a famous tree farm which not only cultivates economical trees, but also services as an ex situ conservation unit for endangered species. Human introduction is therefore a likely source of the disjunctions, but we cannot discount frugivorous bird dispersal, particularly for the individuals in QCS belonging to the Western clade. A ring diversification pattern may have also contributed toward the disjunct nature of QCS (as discussed above).

With the knowledge obtained though population genomics, the best conservation strategy for *P. zhennan* is in situ. Unfortunately, several populations (e.g., QCS, QL, TL, and YC) occur in regions where the human disturbance such as fengshui forestry and tree farming greatly limits available habitat in natural reserves. These reduced habitats can also severely limit gene flow and seedling establishment due to habitat fragmentation and anthropogenic disturbance, a situation that has been observed in case studies of other Chinese Lauraceae such as *Phoebe bournei* (Hemsl.) Yang (Ge, Liu, Shen, & Lin, 2015) and *Neolitsea sericea* (Blume) Koidz. (Wang et al., 2005). *Phoebe zhennan* populations should be protected not only for their genetic diversity but also for their cultural value. Pre‐ and postzygotic barriers may play a vital role in selection against biparental inbreeding, and the relatively low levels of *F*_ST_ within the identified genetic clusters and higher levels of observed heterozygosity suggest that it is vital to monitor factors that facilitate outcrossing in *P. zhennan* such as pollination, seed dispersal and predation, seedling recruitment, and herbivory (Neuschulz, Mueller, Schleuning, & Böhning‐Gaese, 2016).

When ex situ conservation or augmentation is considered, it is crucial that genetic diversity is optimized within the three structure groups, with the mixing of the Western and Eastern structure groups minimized. Translocations using individuals from both Western and Eastern groups may not induce higher genetic diversity, but instead may bring together individuals from groups that cannot readily interbreed. The species will benefit from the continuation of the ban on felling, and while ancient trees are of cultural value, these trees are also likely to decrease the effects of genetic drift and help to increase within‐population genetic diversity while safeguarding the valuable diversity accumulated across past climatic periods.

Finally, the markers obtained in this study could be useful for future forensic work to identify the origin of illegally felled gold‐thread nanmu samples and could be developed further for the policing of illegal trade in closely related species such as *P. bournei*, *P. hui* Cheng ex Yang, and *P. chekiangensis* C.B. Shang.

## 5 CONCLUSIONS

This study used population genomics to evaluate genomic diversity and genetic structure in an endangered plant species (*P. zhennan*) and infer its demographic history. We found that *P. zhennan* displays a pattern of low level of genomic diversity within populations, coupled with three distinct lineages (West1, West2, and East) around the Sichuan Basin, suggesting a long evolutionary history back to the middle Miocene. Geographic isolation‐by‐distance and the historical intensification dynamics of the East Asian monsoon appear to have shaped diversification within *P. zhennan*. These results thus have the potential to establish effective and efficient strategies for the conservation of this endangered species and to improve our understanding of the origin and evolution of plant biodiversity in subtropical China. Coupled with previous research, this study suggests that the East Asian monsoon and geographic distance are important, ongoing influences on the evolution of the floras of subtropical China and potentially East Asia more generally.

## CONFLICT OF INTEREST

The authors declare no conflict of interest.

## AUTHOR CONTRIBUTIONS

**Jian‐Hua Xiao:** Formal analysis (equal); methodology (equal); resources (equal); software (equal); visualization (equal); writing – original draft (equal). **Xin Ding:** Data curation (equal). **Lang Li:** Conceptualization (equal); funding acquisition (equal); project administration (equal). **Hui Ma:** Methodology (supporting). **Xiu‐Qin Ci:** Supervision (supporting); writing – original draft (supporting). **Marlien van der Merwe:** Writing – original draft (equal). **John G. Conran:** Writing – original draft (equal). **Jie Li:** Funding acquisition (lead); project administration (lead); supervision (equal); writing – original draft (equal).

## Supporting information

Supplementary Material

## Data Availability

All relevant genomic data (including complete RAD‐seq loci information) in this study are available under the Data Dryad accession: https://doi.org/10.5061/dryad.7wm37pvnc; the climate data and Maxent input files are uploaded as online supporting information.
