# Supplementary material for: Miocene diversification of a golden‐thread nanmu tree species (Phoebe zhennan, Lauraceae) around the Sichuan Basin shaped by the East Asian monsoon
Source: Ecol Evol. 2020 Aug 26;10(19):10543–57. doi: 10.1002/ece3.6710 (PMC7548194; doi:10.1002/ece3.6710)
Supplement: Supplementary file 1 — Supplementary Material [file ECE3-10-10543-s001.docx]

**Appendix S1**

**Article title:** Miocene diversification of a golden-thread nanmu tree species (*Phoebe zhennan*, Lauraceae) around the Sichuan Basin shaped by the East Asian monsoon

Authors: Jian-Hua Xiao^1,2^, Xin Ding^3^, Lang Li^1,4^, Hui Ma^1^, Xiu-Qin Ci^1,4^, Marlien van der Merwe^5^, John G. Conran^6^, Jie Li^1,4*^

Article acceptance date: Click here to enter a date.

The following Supporting Information is available for this article:

**Fig. S1** Detailed schematics of demographic models 1-20. Models 1-3: divergent with recent and historical gene flow; Models 4-6: divergent without gene flow; Models 7-9: divergent with only historical gene flow. Models 10-12: admixture with recent and historical gene flow; Models 13-15: admixture without gene flow; Models 16-18: admixture with only historical gene flow. Model 19: trifurcation without gene flow; Model 20: trifurcation with gene flow.


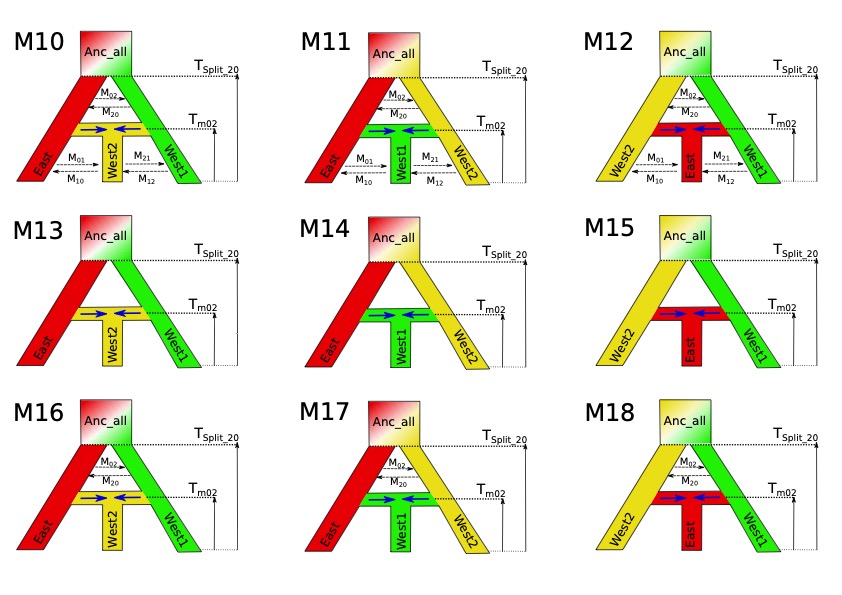

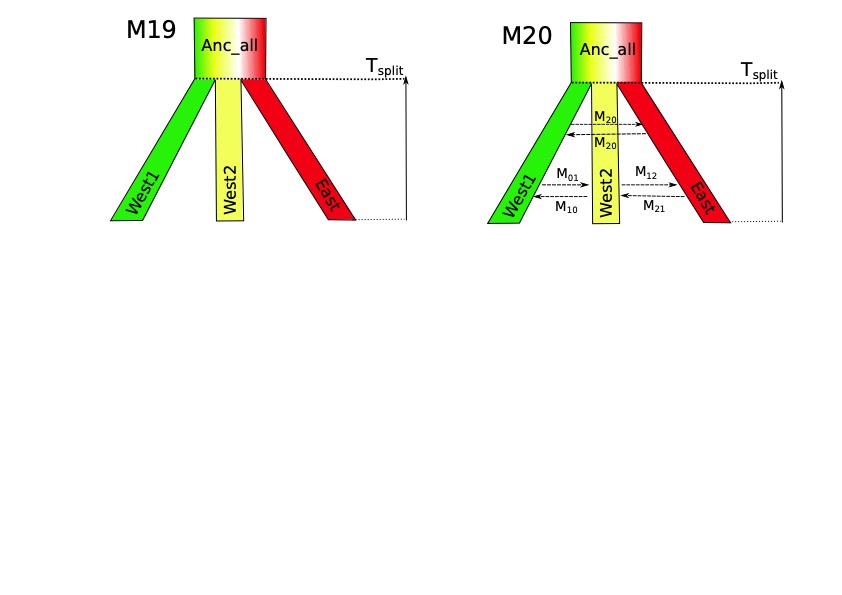


**Fig. S2** Admixture graph of genetic groups from 12 populations obtained with TreeMix. Admixture graphs are shown for models assuming zero and three migration events. The drift parameter reflects the amount of genetic drift that has occurred between populations. Residuals for each migration model are shown below the graph.

**Table S1** Sample sites information of *P. zhennan* used in species distribution modelling (SDM).

| **ID** | **Longitude** | **Latitude** |
| --- | --- | --- |
| 1 | 104.55 | 32.45 |
| 2 | 108.63 | 31.82 |
| 3 | 104.34 | 31.59 |
| 4 | 109.63 | 31.37 |
| 5 | 104.06 | 31.30 |
| 6 | 109.88 | 31.11 |
| 7 | 110.34 | 31.04 |
| 8 | 109.15 | 31.04 |
| 9 | 103.57 | 30.90 |
| 10 | 110.72 | 30.88 |
| 11 | 104.03 | 30.66 |
| 12 | 104.08 | 30.65 |
| 13 | 103.20 | 30.35 |
| 14 | 103.20 | 30.24 |
| 15 | 104.05 | 30.10 |
| 16 | 102.76 | 30.07 |
| 17 | 109.48 | 29.99 |
| 18 | 103.00 | 29.98 |
| 19 | 102.23 | 29.91 |
| 20 | 106.40 | 29.81 |
| 21 | 106.10 | 29.59 |
| 22 | 103.43 | 29.56 |
| 23 | 105.89 | 29.56 |
| 24 | 109.42 | 29.45 |
| 25 | 109.25 | 29.17 |
| 26 | 108.66 | 28.89 |
| 27 | 105.32 | 28.83 |
| 28 | 105.01 | 28.68 |
| 29 | 104.01 | 28.67 |
| 30 | 105.65 | 28.65 |
| 31 | 105.11 | 28.60 |
| 32 | 104.32 | 28.57 |
| 33 | 109.02 | 28.47 |
| 34 | 104.60 | 28.46 |
| 35 | 106.01 | 28.42 |
| 36 | 107.86 | 28.37 |
| 37 | 106.20 | 28.33 |
| 38 | 105.52 | 28.32 |
| 39 | 108.12 | 28.26 |
| 40 | 104.83 | 28.25 |
| 41 | 104.37 | 28.23 |
| 42 | 106.83 | 28.13 |
| 43 | 104.43 | 28.10 |
| 44 | 107.19 | 27.95 |
| 45 | 108.01 | 27.83 |
| 46 | 108.78 | 27.64 |
| 47 | 107.79 | 27.64 |

Table S2 Codes for the 19 environmental variables used in species distribution modelling for *P. zhennan*.

| \| Code \| Variable \| \| --- \| --- \| \| Bio1 \| Annual mean temperature \| \| Bio2 \| Mean diurnal range (mean of monthly (max temp - min temp)) \| \| Bio3 \| Isothermality (Bio2/Bio7) (*100) \| \| Bio4 \| Temperature seasonality (standard deviation *100) \| \| Bio5 \| Max temperature of warmest month \| \| Bio6 \| Min temperature of coldest month \| \| Bio7 \| Temperature annual Range (Bio5-Bio6) \| \| Bio8 \| Mean temperature of wettest quarter \| \| Bio9 \| Mean temperature of driest quarter \| \| Bio10 \| Mean temperature of warmest quarter \| \| Bio11 \| Mean temperature of coldest quarter \| \| Bio12 \| Annual precipitation \| \| Bio13 \| Precipitation of wettest month \| \| Bio14 \| Precipitation of driest month \| \| Bio15 \| Precipitation seasonality (coefficient of variation) \| \| Bio16 \| Precipitation of wettest quarter \| \| Bio17 \| Precipitation of driest quarter \| \| Bio18 \| Precipitation of warmest quarter \| \| Bio19 \| Precipitation of coldest quarter \| |
| --- | --- | --- | --- | --- | --- | --- | --- | --- | --- | --- | --- | --- | --- | --- | --- | --- | --- | --- | --- | --- | --- | --- | --- | --- | --- | --- | --- | --- | --- | --- | --- | --- | --- | --- | --- | --- | --- | --- | --- | --- |

**Table S3** Comparison of demographic models analyzed with fastsimcoal2 for the three lineages of *P. zhennan*.

| Model | MaxEstLhood | k | AIC | ΔAIC |
| --- | --- | --- | --- | --- |
| Model1 | -13597 | 15 | 62651 | 0 |
| Model2 | -13633 | 15 | 62815 | 164 |
| Model3 | -13598 | 15 | 62653 | 2 |
| Model4 | -14146 | 9 | 65161 | 2510 |
| Model5 | -14134 | 9 | 65110 | 2458 |
| Model6 | -14127 | 13 | 65078 | 2426 |
| Model7 | -13677 | 13 | 63008 | 356 |
| Model8 | -14119 | 13 | 65041 | 2034 |
| Model9 | -14117 | 7 | 65031 | 2380 |
| Model10 | -13655 | 7 | 62896 | 245 |
| Model11 | -13675 | 7 | 63010 | 359 |
| Model12 | -13689 | 7 | 63070 | 419 |
| Model13 | -14160 | 7 | 65227 | 2576 |
| Model14 | -14147 | 9 | 65170 | 2519 |
| Model15 | -14118 | 9 | 65038 | 2387 |
| Model16 | -13683 | 9 | 63036 | 385 |
| Model17 | -13726 | 5 | 63235 | 584 |
| Model18 | -14124 | 11 | 65067 | 2416 |
| Model19 | -14124 | 7 | 65059 | 2407 |
| Model20 | -13635 | 9 | 62809 | 158 |

**Method S1** Species distribution modelling (SDM) of *Phoebe zhennan*.

SDM was carried out in Maxent v3.4.1 (Phillips *et al.*, 2006; Elith *et al.*, 2011) to predict the potential and suitable distribution range of the species and also to investigate whether Sichuan basin may have served as a barrier potentially facilitating ring diversification. For occurrence data of *P. zhennan*, we combined our collection records and specimen records Chinese Virtual Herbarium (CVH, http://www.cvh.ac.cn/), National Specimen Information Infrastructure (NSII, <http://www.nsii.org.cn/>) and Global Biodiversity Information Facility (GBIF, http://www. gbif.org/). After removing data for misidentified specimens and spatially duplicated locations (occurrences with an average nearest-neighbor distance of <10 km), a total of 47 occurrence records were retained (Table S1). Nineteen bioclimatic variables (Tables S2) for both Last Glacial Maximum (LGM, ~22, 000 years ago) and present (~1950–2000 year) periods were obtained from the WorldClim database (<http://www.worldclim.org/>; Hijmans *et al.*, 2005).

To avoid multicollinearity, the initial variables were filtered based on the results of Pearson’s correlation analysis (Synes & Osborne, 2011). For each highly correlated variable pair (Pearson’s *r*≥0.7), the variable that gave a higher value in the regularized gain and the percent contribution to the Maxent model was retained. The environmental variables finally included in the models were: annual mean temperature (Bio1), temperature seasonality (Bio4), max temperature of the warmest month (Bio5), annual precipitation (Bio12), precipitation seasonality (Bio15), precipitation of the warmest quarter (Bio18) and precipitation of the coldest quarter (Bio19).

The Maxent was configured with 75% of species presence data for training and 25% for testing data and sed mostly default parameters, including regularization=1, maximum iteration=500, convergence threshold=10^-5^, maximum number of background points=10000. Sampling procedure was replicated 50 times. The area under the curve (AUC) was estimated to test the accuracy of the model prediction, with AUC values interpreted as excellent (AUC>0.9), good (0.9 >AUC>0.8), fair (0.8>AUC>0.7), poor (0.7>AUC>0.60) and fail (0.6>AUC>0.5) (Swets, 1988). SDM analyses were carried out at a resolution of 2.5 arc-min. The distributions of these periods were then plotted on a Chinese map using a geographic information system as implemented in the software ArcGIS 10.4 (Environmental Systems Research Institute, Inc.)

**Method S2** Demographic history inference for *P. zhennan*

The demographic history of the *P. zhennan* was reconstructed using *fastsimcoal2* (Excoffier *et al.*, 2013)*.* According to STRUCTURE, PCA and TreeMix analyses, we tested 20 models of three lineages. Testing model includes three alternative scenarios of lineage divergence and two migration matrices (total absence of post-divergence gene flow and a full migration matrix of asymmetric gene flow) (Figure S1). We estimated the composite likelihood of the observed data given a specified model using the site frequency spectrum (SFS). For simulations, 12 individuals for west1 sub-lineage, 12 individuals for west2 sub-lineage, and 15 individuals for east lineage. Using 16, 210 SNPs were called for a folded joint SFS file. We assumed a mutation rate of 1.5e-8 per site per generation. A generation time of 15 years was accepted when converting estimates to units of years.

Each model was run 50 replicates considering 40 ECM cycles, 100,000 simulations for the calculation of the composite likelihood. For using of the AIC allowed for model comparison despite varying numbers of parameters, model comparison was based on the using the Akaike information criterion and Akaike’s weight of evidence. AIC scores were calculated for each model using the formula: [(2k)–(2×ln10×MOL)], where k is the number of parameters included in model.

AIC scores subtracted from the best-fit model to give a ∆AIC value (AIC~[0,2], with substantial modeling and the best model has AIC=0).

Finally, we estimated 95% confidence intervals (CIs) of parameter estimates from100 bootstrap replicates by simulating the SFS from the *_maxL.par file and re-estimating parameters each time. Model scoring supported the most likely scenario (Model 1, Table S3) of the initial split of east from the putative ancestor and followed divergence between West1 and West2 sub-lineages.
